# Initial-Visit Specialty Triage in Rare Diseases Using Large Language Models: Retrospective Benchmarking Study

**DOI:** 10.2196/101711

**Published:** 2026-07-23

**Authors:** Jie Song, Zhichuan Xu, Meng Xiao, Cheng Bi, Yuxin Zhang, Xin Zheng, Xiaoran Li, Qiongfang Cao, Ziyu Lu, Hao Yang, Bairong Shen

**Affiliations:** 1 Department of Ophthalmology and Institutes for Systems Genetics, Frontiers Science Center for Disease-related Molecular Network, West China Hospital of Sichuan University, No.2222, Xinchuan Road, Gaoxin District, Chengdu, Sichuan, 610041, China, 86 15995854635; 2 Department of Computer Science and Information Technologies, Iberian Society of Telehealth and Telemedicine, Universidade da Coruña, A Coruña, Galicia, Spain

**Keywords:** rare diseases, large language models, specialty triage, artificial intelligence, digital health

## Abstract

**Background:**

Specialty triage at first contact is an overlooked step in early diagnostic pathways for rare diseases. Patients often present with overlapping, multisystem, and atypical manifestations, making first-visit specialty selection challenging and potentially prolonging diagnostic pathways.

**Objective:**

The aim of this study is to evaluate the accuracy, response time, and consistency of large language models (LLMs) for initial-visit specialty triage in rare diseases across multiple datasets, and to compare their performance with registered nurses and nonmedical participants.

**Methods:**

In this retrospective benchmarking study, we used 5 rare disease datasets: a publication-derived case set, 3 RareBench-derived datasets, and a Facial phenotype-Gene-Disease Dataset-derived set. Fourteen LLMs were evaluated over 5 independent runs per case. Performance was assessed using accuracy, response time, and consistency, with subgroup analyses by model accessibility, reasoning mode, parameter scale, and phenotype count. Human comparison was conducted on the publication-derived case set using registered nurses and nonmedical participants.

**Results:**

Across datasets, model accuracy ranged from 0.4378 to 0.7141. Claude-opus-4‐5 achieved the highest accuracy (0.7141) and consistency (0.9653), averaging 10.79 seconds per case. GPT-5.1 had the shortest response time (3.39 s/case) and high accuracy (0.6948). Proprietary models had numerically higher average accuracy than open-weight models (0.6973 vs 0.6365). Nonthinking models achieved higher average accuracy than thinking models (0.6789 vs 0.5826) and had shorter response times, although this exploratory comparison was based on a small number of thinking models. Accuracy varied by phenotype count, with higher performance in cases with 1 to 2 or more than 14 phenotypes. On the publication-derived case set, LLMs achieved higher average accuracy than registered nurses and nonmedical participants (0.5978 vs 0.4914 and 0.4573).

**Conclusions:**

LLMs showed potential as assistive tools for initial-visit specialty triage in rare diseases. Model choice, reasoning mode, and phenotype information density influenced performance, but subgroup findings should be interpreted cautiously. Future work should evaluate LLM-based specialty triage in prospective clinical settings and develop clinician-supervised workflows with traceable evidence support.

## Introduction

In the diagnosis journey of rare diseases, specialty triage at the initial visit (ie, the process by which patients are initially directed to a medical specialty) is a frequently overlooked yet crucial step. The clinical manifestations of most rare diseases substantially overlap with those of common diseases [1,2]. Consequently, patients usually first seek medical attention in nonspecialist settings. In countries with gatekeeping systems [3] (eg, the United Kingdom, the Netherlands, Canada), this triage is primarily conducted by general practitioners, who are responsible for initial assessment and referral; in systems where patients can directly access specialty care (eg, China), it typically occurs in general hospitals, where triage nurses or intake physicians direct patients to specific specialty clinics.

However, the training content and clinical experience of these health care providers [4], as well as the design of most existing electronic triage tools [5-7], are predominantly oriented toward common, high-prevalence diseases rather than rare diseases. Under conditions of limited information, health care providers must nonetheless make an initial judgment about the most appropriate specialty to which the patient should be referred. Whether patients can be accurately directed to the relevant specialty at the initial visit largely determines the efficiency of subsequent diagnosis and treatment and is critically important for shortening diagnostic delays [8].

In this context, inappropriate specialty triage has become an important source of diagnostic delay for patients with rare diseases. In real-world clinical practice, patients are often directed to inappropriate specialties, leading to multiple transfers between specialties and repeated misdiagnoses. Existing evidence [9] indicates that 22% of patients consulted at least 8 health care professionals, 73% experienced at least 1 misdiagnosis, and the average diagnostic delay was as long as 4.7 years. This prolonged and convoluted diagnostic process further increases the burden on patients and their families, as well as the costs to the health system [10].

In recent years, large language models (LLMs) have demonstrated unprecedented capabilities in natural language processing and generation, and have shown promising performance in medical question answering and diagnostic support [11-13]. However, existing LLM-based medical applications have largely focused on emergency triage [14-16] (assigning patients to different urgency levels), with very little attention given to specialty triage (directing patients to the appropriate specialty). In particular, applications in populations with rare diseases remain unexplored.

Therefore, in this paper, we evaluated the performance of LLMs in specialty triage for rare diseases, a clinically important yet underexplored step in the early diagnostic pathway. We evaluated rare disease specialty triage using 5 datasets: a curated publication-derived case set, 3 datasets adapted from RareBench [17], and a dataset derived from the Facial phenotype-Gene-Disease Dataset (FGDD) [18]. Each case was adapted to reflect information typically available at the initial visit, with the task defined as selecting the most appropriate first-visit specialty. We conducted 4 analyses: overall performance across 14 LLMs using accuracy, response time, and consistency; comparisons by model type and parameter scale, including proprietary vs open-weight models, thinking vs standard models, and models of different sizes; phenotype-count stratification to assess the impact of clinical information volume; and comparison with registered nurses and nonmedical participants on the publication-derived case set. The aim of this study was to determine whether LLMs can serve as reliable decision-support tools for the first specialty referral of patients with rare diseases, particularly when only limited initial-visit clinical information is available, and to clarify which model- and case-level factors shape triage performance.

## Methods

### Dataset

We included 5 rare disease datasets: a publication-derived case set, 3 RareBench-derived datasets (RareBench_HMS, RareBench_LIRICAL, and RareBench_MME), and an FGDD-derived set. The sample size and number of diseases in each dataset are shown in Table 1.

**Table 1.** Summary of the datasets included in this study.

Dataset	Sample size, n	Disease, n	Top 3 disease specialty distributions, n/N (%)
Publication set	78	77	Rheumatology and Immunology (26/78, 33.3%) Neurology (24/78, 30.8%) Hematology (16/78, 20.5%)
RareBench HMS^a^	83	24	Rheumatology and Immunology (72/83, 86.7%) Hematology (72/83, 86.7%) Ophthalmology and Orthopedic Surgery (4/83, 4.8%)
RareBench LIRICAL^b^	330	221	Neurology (230/330, 69.7%) Pediatrics (169/330, 51.2%) Endocrinology (139/330, 42.1%)
RareBench MME^c^	38	16	Pediatrics (26/38, 68.4%) Neurology (25/38, 65.8%) Endocrinology (23/38, 60.5%)
FGDD^d^	100	100	Otolaryngology-Head and Neck Surgery (78/100, 78%) Pediatrics (75/100, 75%) Endocrinology (38/100, 38%)

a HMS: Hannover Medical School dataset.

b LIRICAL: Likelihood Ratio Interpretation of Clinical Abnormalities dataset.

c MME: Matchmaker Exchange dataset.

d FGDD: Facial phenotype-Gene-Disease Dataset.

The publication set was constructed from published rare disease case reports. Each case was converted into an initial-visit clinical summary containing information available at first contact, including demographics, chief complaint, clinical manifestations, medical history, and family history. The PubMed identifiers of included reports are provided in Table S1 in Multimedia Appendix 1.

The 3 RareBench-derived datasets [17] were adapted from rare disease diagnosis datasets for the specialty triage task. Human Phenotype Ontology [19] phenotype terms were converted into natural-language descriptions, and phenotype information was filtered to retain findings obtainable at the initial visit. The FGDD-derived set [18] focused on facial phenotypes, which are typically observable during first contact; 100 diseases were sampled at the disease level, with 1 case retained per disease.

For all datasets, the reference answer was defined as the appropriate first-visit specialty. Two clinical reviewers with more than 5 years of clinical experience assigned reference labels by consensus using Orphanet [20] disease classifications, Orphanet disease descriptions, and case symptoms. Because Orphanet classifications reflect organ-system involvement and disease-category information closely related to specialty relevance, they provided a structured and clinically grounded basis for assigning first-visit specialties.

Because rare diseases can involve multiple organ systems, each case could have more than 1 acceptable specialty. A prediction was considered correct if it matched any acceptable reference specialty. All model and human evaluations used the same predefined list of 30 candidate specialties, provided in Table S2 in Multimedia Appendix 1. The 5 datasets differed in source, case structure, and specialty distribution. These differences should be considered when interpreting dataset-specific descriptive results.

### LLMs Configuration and Evaluation

We evaluated 14 API-accessed LLMs from the Claude, Gemini, GPT, Qwen3, LLaMA-3.1, and DeepSeek families, including both proprietary and open-weight models. Model details are provided in Table 2, and the exact API model identifiers and API access period are provided in Table S3 in Multimedia Appendix 1. Temperature was set to 0.3 for all calls. Each model received a structured triage prompt with patient information, the same candidate specialty list, and an instruction to select the most appropriate first-visit specialty. RareBench_HMS, RareBench_LIRICAL, RareBench_MME, and FGDD used the same phenotype-based prompt template, whereas the publication set used a clinical-summary template with demographics, clinical manifestations, medical history, and family history. Both templates are provided in Table S4 in Multimedia Appendix 1.

**Table 2.** Summary of the large language models evaluated in this study.

LLM^a^	Parameters	Active parameters	Open weights
Claude-opus-4‐5	Not disclosed	Not disclosed	✕
Gemini-3-pro	Not disclosed	Not disclosed	✕
GPT-5	Not disclosed	Not disclosed	✕
GPT-5-mini	Not disclosed	Not disclosed	✕
GPT-5.1	Not disclosed	Not disclosed	✕
Qwen3-235B-A22B-Instruct	235B	22B	✓
Qwen3-235B-A22B-Thinking	235B	22B	✓
Qwen3-30B-A3B-Instruct	30B	3B	✓
Qwen3-30B-A3B-Thinking	30B	3B	✓
LLaMA-3.1-8B	8B	0B	✓
LLaMA-3.1-70B	70B	0B	✓
LLaMA-3.1-405B	405B	0B	✓
DeepSeek-V3.2	671B	37B	✓
DeepSeek-R1	671B	37B	✓

a LLM: large language model.

Each case was evaluated in 5 independent runs. We recorded response time, extracted the selected specialty, and calculated accuracy against the reference specialties. Overall performance was calculated by pooling all cases across datasets, making accuracy weighted by dataset size; larger datasets such as RareBench_LIRICAL contributed proportionally more to the estimate. Consistency was defined as the proportion of repeated runs matching the modal prediction for each case, reflecting output stability across repeated evaluations; for example, if the 5 predictions for a case were “Neurology, Neurology, Ophthalmology, Neurology, Ophthalmology,” the modal answer would be “Neurology,” and the consistency score would be 3/5=0.60. For the phenotype-count analysis, we included cases with explicit phenotype annotations from RareBench_HMS, RareBench_LIRICAL, RareBench_MME, and FGDD. Five data-driven strata with approximately comparable sample sizes were used: 1‐2 phenotypes (n=116), 3‐5 (n=114), 6‐8 (n=103), 9‐13 (n=116), and ≥14 (n=102).

### Human Evaluators

The human comparison experiment was conducted on the publication set as an exploratory human-reference comparison. Six participants were included and independently completed the same specialty triage task as the LLMs. Using the same case descriptions and the same list of candidate specialties, each participant submitted 1 final specialty answer for each case. We included equal numbers of registered nurses and nonmedical participants to provide a balanced comparison under the same case materials, candidate specialty list, and scoring rules. The nurse group included 3 registered nurses, all with more than 5 years of clinical experience and experience with routine clinical triage or patient guidance; however, they had limited direct exposure to rare disease cases and no specialized training in rare disease triage. The nonmedical group included 3 participants with bachelor’s degrees in computer science and no medical education or clinical training. The nurse group was intended to approximate a nonspecialist clinical intake baseline, whereas the nonmedical group served as a lower-bound reference for specialty selection without medical training.

Human responses were evaluated using the same reference answers and scoring rules as those used for the LLMs. A response was considered correct if the selected specialty matched any acceptable reference specialty label for that case. We also recorded the total time taken by each participant to complete all cases and used it to calculate the average response time per case for comparison with model’s response time.

### Statistical Analysis

Model accuracy and response time were reported as mean (SD) across 5 independent runs. Consistency was summarized as the mean across cases, with SD calculated across case-level consistency scores. For model accuracy, 95% CIs were estimated using clustered case-level bootstrap resampling, in which cases were resampled with replacement and repeated runs for each sampled case were kept together.

Subgroup comparisons were performed at the model level using mean differences and exact permutation tests. Human-LLM accuracy comparisons used case-level mean accuracy and paired bootstrap confidence intervals with paired permutation tests. Human-LLM response-time comparisons were interpreted as exploratory because timing was measured differently for humans and APIs.

### Ethical Considerations

This study was not submitted to an ethics committee for review. The study used legally obtained, publicly available rare disease case reports and public benchmark datasets containing anonymized or deidentified information. According to Article 32, item (1), of the *Measures for Ethical Review of Life Science and Medical Research Involving Humans* issued by the National Health Commission of the People’s Republic of China [21,22], research using legally obtained public data, or data generated through observation without interfering with public behavior, may be exempted from ethics review. Therefore, under this policy, not applying for ethics board review was appropriate, and no study protocol or application number is applicable.

## Results

### Overall Performance Comparison Across LLMs

**Figure 1.**
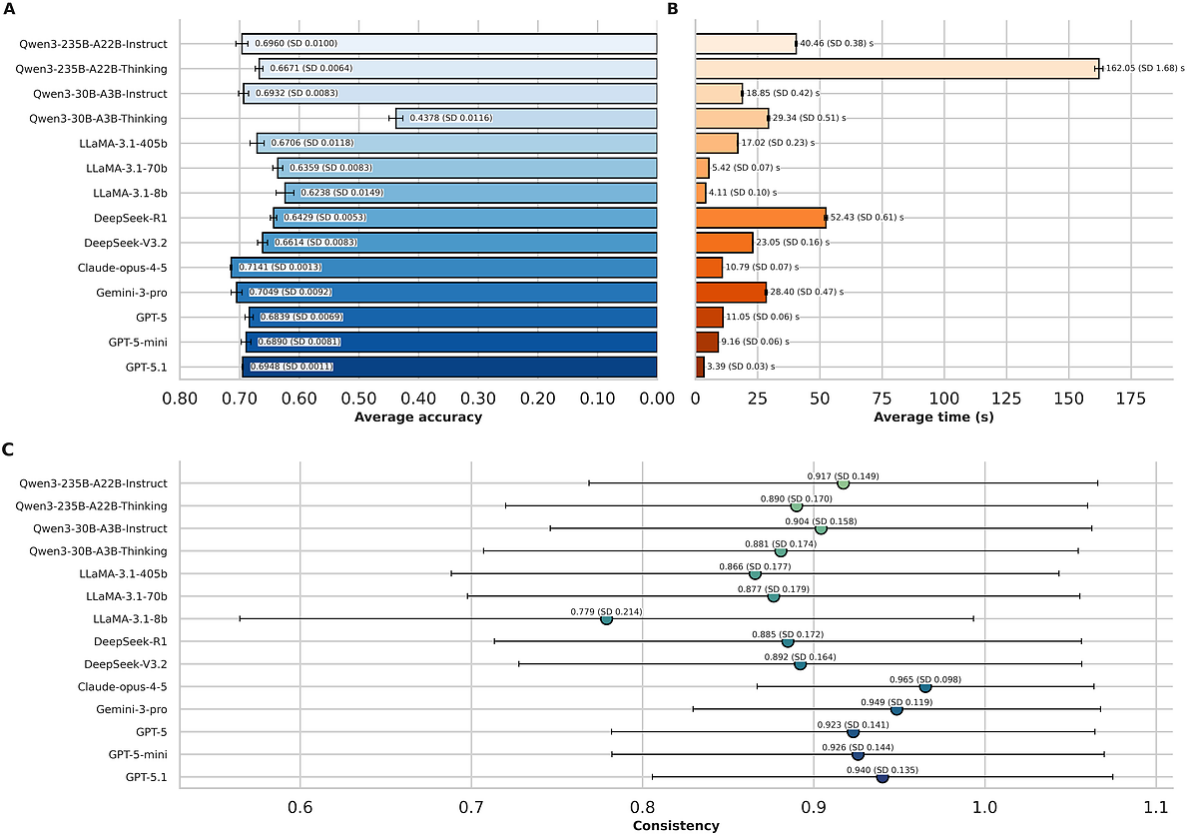
Overall performance of large language models for rare disease specialty triage across all datasets. (A) Average accuracy across 5 independent runs. (B) Average response time per case. (C) Average consistency across 5 repeated runs. Overall accuracy and response time were calculated by pooling all cases, so estimates were weighted by dataset size.

Figure 1 shows the overall performance of the 14 LLMs across the 5 datasets, including accuracy, response time, and consistency. Detailed results for each individual dataset are provided in Figures S1-S5 in Multimedia Appendix 1. Accuracy and response time are presented as mean (SD) across 5 independent runs, and consistency is presented as mean (SD) across cases. Clustered case-level bootstrap 95% CIs for model accuracy are provided in Table S5 in Multimedia Appendix 1.

In Figure 1A, overall accuracy varied substantially across models, ranging from 0.4378 to 0.7141. Claude-opus-4‐5 achieved the highest average accuracy (0.7141), followed by Gemini-3-pro (0.7049), Qwen3-235B-A22B-Instruct (0.6960), GPT-5.1 (0.6948), and Qwen3-30B-A3B-Instruct (0.6932). Qwen3-30B-A3B-Thinking had the lowest accuracy (0.4378).

In Figure 1B, GPT-5.1 had the shortest overall average response time (3.39 s/case), followed by LLaMA-3.1-8B (4.11 s/case), LLaMA-3.1-70B (5.42 s/case), GPT-5-mini (9.16 s/case), and Claude-opus-4‐5 (10.79 s/case). Qwen3-235B-A22B-Thinking had the longest response time (162.05 s/case), followed by DeepSeek-R1 (52.43 s/case) and Qwen3-235B-A22B-Instruct (40.46 s/case). GPT-5.1 combined high accuracy (0.6948) with the shortest response time, while Claude-opus-4‐5 achieved the highest accuracy with moderate response time.

In Figure 1C, consistency reflected the stability of model answers across the 5 repeated runs. Claude-opus-4‐5 achieved the highest majority-vote consistency (0.9653), followed by Gemini-3-pro (0.9485), GPT-5.1 (0.9402), GPT-5-mini (0.9259), and GPT-5 (0.9231). LLaMA-3.1-8B had the lowest majority-vote consistency (0.7790). Models with higher accuracy generally also showed higher consistency across repeated runs.

Across the 3 metrics, Claude-opus-4‐5 showed the strongest overall performance, with the highest accuracy (0.7141), the highest consistency (0.9653), and an average response time of 10.79 seconds per case. GPT-5.1 achieved an accuracy of 0.6948 with an average response time of 3.39 seconds per case, indicating high efficiency. Some reasoning models showed limited performance gains relative to their time cost. For example, Qwen3-235B-A22B-Thinking had an average response time of 162.05 seconds per case, with an accuracy of 0.6671 and a consistency of 0.890.

### Performance Stratified by Model Type, Reasoning Mode, Phenotype Count, and Parameter Scale

**Figure 2.**
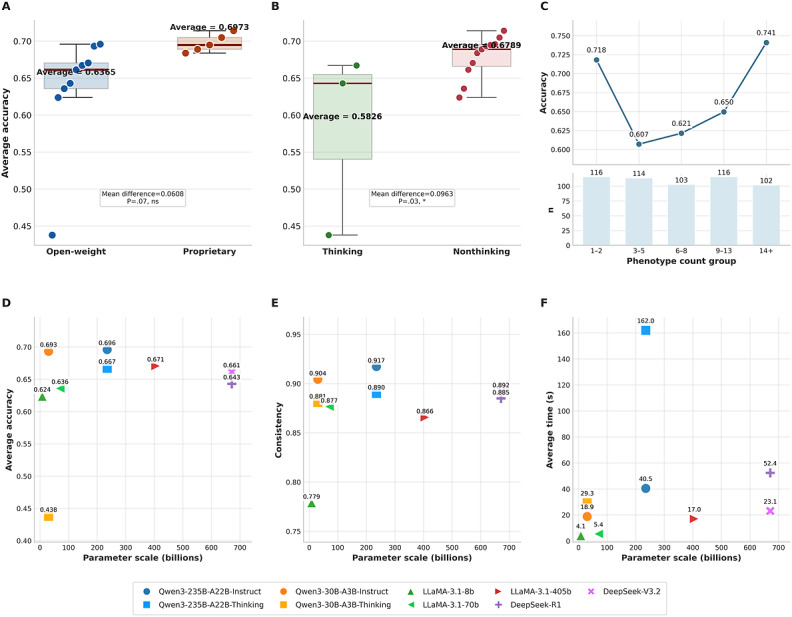
Performance comparison by model type, reasoning mode, phenotype number, and parameter scale. (A) Accuracy of open-weight vs proprietary models. This analysis included 551 cases from RareBench_HMS, RareBench_LIRICAL, RareBench_MME, and Facial phenotype-Gene-Disease Dataset with explicit phenotype annotations. (B) Accuracy of thinking vs nonthinking models. (C) Accuracy across phenotype-count strata. (D) Accuracy of open-weight models by parameter scale. (E) Consistency of open-weight models by parameter scale. (F) Response time of open-weight models by parameter scale. Statistical significance is denoted as ns, not significant; *P<.05; **P<.01; ***P<.001.

The subgroup comparison results are shown in Figure 2. Model type, reasoning mode, parameter scale, and the number of phenotypes in each case all influenced model performance in specialty triage.

For model accessibility (Figure 2A), proprietary models achieved higher average accuracy than open-weight models (0.6973 vs 0.6365). Among proprietary models, Claude-opus-4‐5 and Gemini-3-pro achieved average accuracies of 0.7141 and 0.7049, respectively. Among open-weight models, Qwen3-235B-A22B-Instruct (0.6960) and Qwen3-30B-A3B-Instruct (0.6932) performed best, reaching a level close to several proprietary models. However, this model-level exploratory comparison was not statistically significant (proprietary n=5 vs open-weight n=9; mean difference=0.0608; exact permutation *P*=.07).

For reasoning mode (Figure 2B), nonthinking models achieved an average accuracy of 0.6789, compared with 0.5826 for thinking models. This pattern was also observed within the same model family: Qwen3-235B-A22B-Instruct outperformed Qwen3-235B-A22B-Thinking (0.6960 vs 0.6671), and Qwen3-30B-A3B-Instruct substantially outperformed Qwen3-30B-A3B-Thinking (0.6932 vs 0.4378). In terms of response time, thinking models also showed longer latency. Qwen3-235B-A22B-Thinking and Qwen3-30B-A3B-Thinking had average response times of 162.05 and 29.34 seconds per case, respectively, both longer than their corresponding Instruct versions. The model-level difference favored nonthinking models over thinking models (n=11 vs n=3; mean difference=0.0963; exact permutation *P*=.03). However, this subgroup analysis should be interpreted cautiously because it was based on a small number of models and represents an exploratory model-level ecological comparison.

In the phenotype-count analysis (Figure 2C), 551 cases from the phenotype-based datasets were divided into 5 data-driven strata with approximately comparable sample sizes: 1‐2 phenotypes (n=116), 3‐5 phenotypes (n=114), 6‐8 phenotypes (n=103), 9‐13 phenotypes (n=116), and ≥14 phenotypes (n=102). These sample sizes are shown by the bar plot in Figure 2C. Accuracy showed an exploratory nonlinear pattern, with higher accuracy in the 1‐2 phenotype group (0.718) and the ≥14 phenotype group (0.741), and lower accuracy in the intermediate phenotype-count groups. One possible explanation is that cases with very few phenotypes may represent more distinctive rare diseases with a clearer specialist match, whereas intermediate-count cases may involve multisystem presentations with more ambiguous specialty assignment. As phenotype counts rise further, richer clinical information appears to support more accurate triage decisions. Therefore, this analysis should be interpreted as descriptive and hypothesis-generating rather than confirmatory.

In the parameter-scale analysis (Figure 2D-F), open-weight models showed nonlinear associations between parameter scale and accuracy, consistency, and response time. For accuracy (Figure 2D), Qwen3-30B-A3B-Instruct achieved 0.6932, close to Qwen3-235B-A22B-Instruct (0.6960) and higher than LLaMA-3.1-405B (0.6706) and DeepSeek-V3.2 (0.6614). For consistency (Figure 2E), stability generally improved with scale, increasing from 0.779 for LLaMA-3.1-8B to 0.866 for LLaMA-3.1-405B and 0.917 for Qwen3-235B-A22B-Instruct. However, DeepSeek-R1 and DeepSeek-V3.2 reached only 0.885 and 0.892 despite their 671B scale. For response time (Figure 2F), larger models generally required longer inference, especially in reasoning mode: Qwen3-235B-A22B-Thinking took 162.05 seconds per case vs 40.46 seconds per case for its Instruct counterpart, and DeepSeek-R1 took 52.43 seconds per case vs 23.05 seconds per case for DeepSeek-V3.2. Smaller LLaMA models were faster, with 4.11 seconds per case for 8B and 5.42 seconds per case for 70B. Overall, rare disease triage performance depended jointly on model scale, architecture, and reasoning paradigm.

### Compare With Human Evaluators

**Figure 3.**
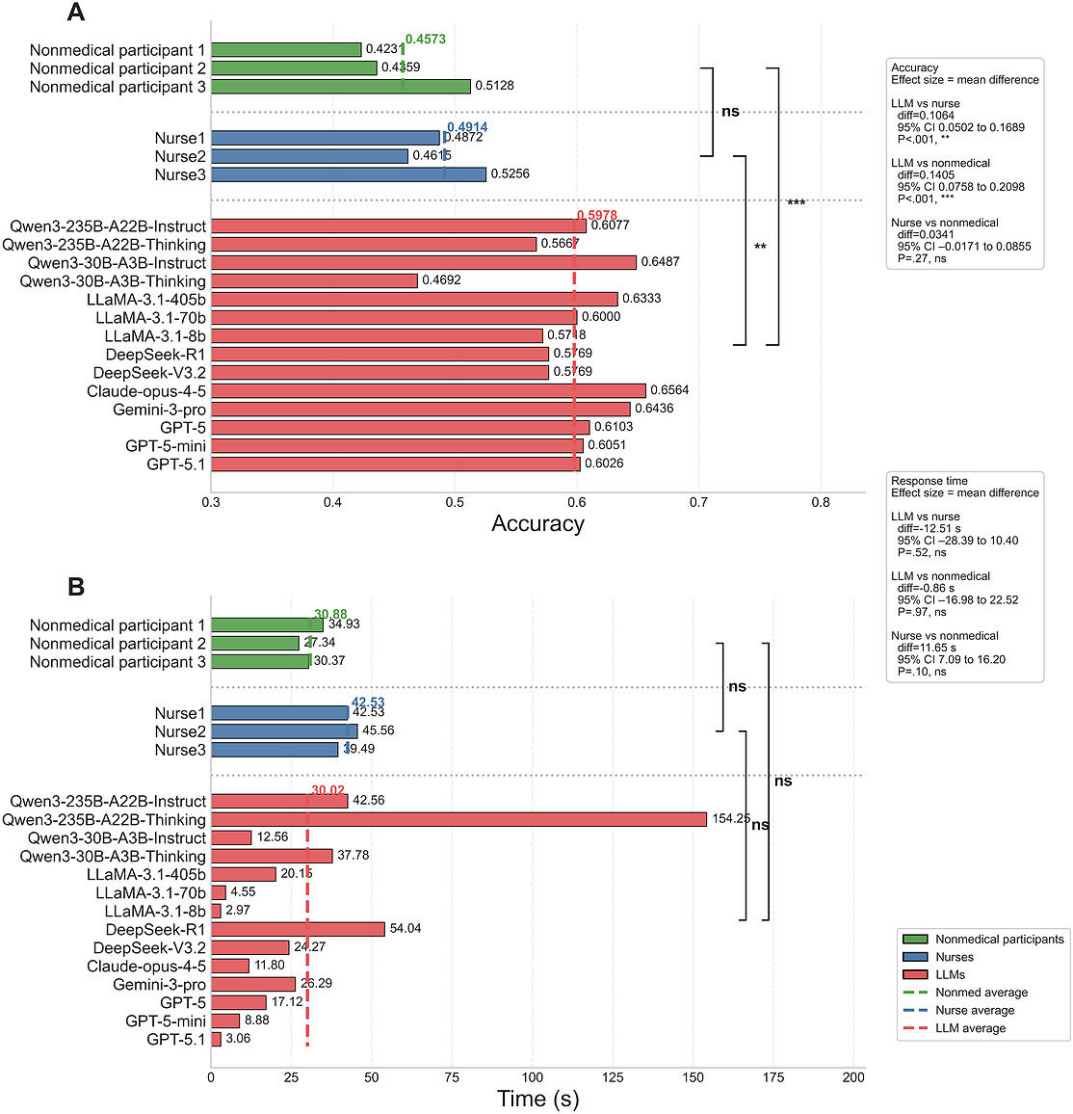
Comparison of large language models (LLMs) with human participants on the publication set. (A) Accuracy of nonmedical participants, registered nurses, and LLMs. (B) Average response time per case for nonmedical participants, registered nurses, and LLMs. Statistical significance is denoted as ns, not significant; *P<.05; **P<.01; ***P<.001.

The results of the human comparison experiment are shown in Figure 3. This evaluation was conducted only on the publication set.

In Figure 3A, the LLM group achieved higher average accuracy (0.5978) than the nurse group (0.4914) and nonmedical participant group (0.4573). Among the 14 LLMs, 13 exceeded the nurse-group average, and all exceeded the nonmedical group average. Claude-opus-4‐5 performed best on the publication set (0.6564), followed by Qwen3-30B-A3B-Instruct (0.6487) and Gemini-3-pro (0.6436). The best-performing individual nurse and nonmedical participant achieved accuracies of 0.5256 and 0.5128, respectively. Case-level paired comparisons showed that LLMs significantly outperformed nurses (mean difference=0.1064, 95% CI 0.0502-0.1689; *P*<.001) and nonmedical participants (mean difference=0.1405, 95% CI 0.0758-0.2098; *P*<.001), whereas nurses and nonmedical participants did not differ significantly (mean difference=0.0341, 95% CI −0.0171 to 0.0855; *P*=.27).

In Figure 3B, the LLM group had an average response time of 30.02 seconds per case, compared with 42.53 seconds per case for nurses and 30.88 seconds per case for nonmedical participants. Among the 14 LLMs, 11 were faster than the nurse-group average, and 10 were faster than the nonmedical group average. LLaMA-3.1-8B and GPT-5.1 were the fastest models, at 2.97 and 3.06 seconds per case, respectively, while Qwen3-235B-A22B-Thinking was the slowest, at 154.25 seconds per case. Response-time comparisons showed no statistically significant differences between LLMs and nurses (mean difference=−12.51 s, 95% CI −28.39 to 10.40; *P*=.52), LLMs and nonmedical participants (mean difference=−0.86 s, 95% CI −16.98 to 22.52; *P*=.97), or nurses and nonmedical participants (mean difference=11.65 s, 95% CI 7.09 to 16.20; *P*=.10). Because human response time was measured as total session time divided by case count, whereas LLM response time was measured per API call, and because API response time may be affected by network conditions, server-side load, and available computing resources, these timing results should be interpreted as exploratory coarse references rather than directly equivalent efficiency comparisons.

## Discussion

### Principal Findings

This study systematically evaluated LLMs for initial-visit specialty triage in rare diseases using 14 models, 5 datasets, and 6 human comparison participants. LLMs achieved relatively high accuracy in this complex task, with Claude-opus-4‐5 showing the strongest overall performance: average accuracy of 0.7141 and consistency of 0.9653. Better-performing models generally produced both higher accuracy and more stable answers across repeated runs. Thinking models showed lower mean accuracy and higher time costs in this setting. Phenotype count had a nonlinear relationship with triage accuracy, with better performance in cases containing very few or abundant phenotypes and weaker performance in intermediate-count cases. On the publication set, LLMs achieved higher overall accuracy than both nurse and nonmedical participant groups; response-time results should be treated as exploratory coarse references because human time was estimated from total session time per case, while LLM time was measured per API call. These findings support the potential value of LLMs as assistive tools for initial-visit specialty triage in rare diseases.

### Model Performance and Clinical Implications

In terms of overall performance, Claude-opus-4‐5 achieved an average accuracy of 0.7141 and a consistency of 0.965, indicating that rare disease specialty triage can be performed reliably by LLMs. The better-performing models produced highly stable answers across repeated runs, a property that is crucial for clinical deployment because clinicians need consistent decision support in real-world use. At the same time, the upper accuracy of 0.7141 indicates that 28.6% (899/3145) of cases were still directed to an incorrect specialty. Therefore, at the current stage, LLMs are better positioned as assistive tools for initial triage, providing candidate specialty suggestions, with their outputs interpreted alongside clinical judgment.

### Reasoning Mode and Phenotype Information Density

In exploratory model-level subgroup analyses, thinking models showed limited advantage in this study, with lower mean accuracy than nonthinking models (0.5826 vs 0.6789). This finding should be interpreted cautiously because only 3 thinking models were included. Therefore, the observed lower performance may reflect a combination of reasoning mode, model family, parameter scale, and model-specific behavior rather than the effect of thinking mode alone. Within these model families, Qwen3-235B-A22B-Instruct outperformed its Thinking counterpart (0.6960 vs 0.6671), as did Qwen3-30B-A3B-Instruct (0.6932 vs 0.4378). Prior work on rare disease diagnosis reported reduced performance with chain-of-thought prompting [23]. This related finding provides background for interpreting our observation that thinking-mode models showed lower performance in rare disease specialty triage. Rare disease specialty triage is a knowledge-intensive task. When disease-phenotype knowledge is insufficient, multiple-step reasoning cannot bridge the knowledge gap; instead, it may amplify error signals along the reasoning chain. At the same time, we agree that the broader concern may also be relevant to human judgment: in initial rare disease triage, humans may need to make decisions from incomplete, atypical, or multisystemic information, and limited rare disease knowledge may allow early misinterpretations to influence subsequent decisions [24,25].

The phenotype-count analysis suggested a nonlinear descriptive pattern. Cases with only 1‐2 phenotypes may sometimes contain highly distinctive clues that map directly to a specialist, whereas cases with intermediate phenotype counts may include overlapping multisystem manifestations that make specialty assignment more ambiguous. In contrast, cases with many phenotypes may provide enough contextual information for models to infer a more stable specialty direction. These findings should be interpreted cautiously and require validation in future datasets.

### Comparison With Previous Work

Compared with previous studies, this study focuses on a clinical step that has received limited systematic evaluation. Existing LLM-based triage studies have mainly focused on urgency triage, in which the task is to determine the patient’s urgency level [14,26]. Existing LLM studies in rare diseases have focused more on diagnostic assistance [27] or knowledge-based question answering [28]. Conventional department recommendation tools and outpatient triage systems have mainly targeted common diseases and high-prevalence symptoms [5-7], with limited optimization for the multisystemic, atypical, and long-tail phenotypes of patients with rare diseases. This study proposes and evaluates rare disease specialty triage as an application scenario for LLMs, providing an upstream decision-support direction for reducing diagnostic delay in rare diseases.

### Deployment Considerations

The experimental results suggest 2 practical deployment pathways. First, open-weight models can be deployed within hospital intranets or regulated medical environments. Qwen3-235B-A22B-Instruct achieved an accuracy of 0.6960, approaching the strongest proprietary models while supporting medical data privacy requirements. Second, GPT-5.1 achieved an accuracy of 0.6948 with a response time of 3.39 seconds per case, showing strong potential for real-time triage assistance.

### Limitations

This study has several limitations. First, the task used a predefined list of 30 candidate specialties, which simplified real-world specialty triage and may not fully reflect the specialty structure or triage workflow of a single health care system. The unified list was necessary for standardized model comparison across datasets, but actual clinical application would require adaptation to the target institution or health care system. Second, reference-label uncertainty was not formally quantified. Reference labels were assigned through a 2-reviewer consensus rather than through independent annotation by a larger expert panel. Therefore, interrater reliability metrics such as Cohen κ could not be calculated. Although multiple acceptable first-visit specialties were allowed for each case, residual uncertainty in the reference standard may still affect accuracy estimates and model ordering, especially when differences between closely performing models are very small. For example, small gaps such as 0.6948 versus 0.6960 should be interpreted cautiously because they may be sensitive to reference-label uncertainty. Third, the human comparison included only 6 participants, including nurses and nonmedical participants, providing a limited human-reference baseline. This sample size is too small for major claims about human-LLM performance differences across broader clinical populations, and the results should therefore be interpreted as exploratory. In addition, matching one predefined acceptable specialty evaluates one specific component of triage performance, while real-world triage quality also depends on handling ambiguous symptoms, eliciting missing information, and prioritizing among multiple reasonable specialties. Fourth, cases were based on structured text summaries or curated phenotype descriptions, which may simplify the complexity of real initial-visit presentations. Rare disease phenotypes are often overlapping, multisystemic, and atypical, and patients’ complete self-reports may contain richer context, symptom chronology, and subjective descriptions than structured summaries or ontology-derived phenotype labels. Fifth, the task mainly used English case information, so cross-lingual generalizability requires further validation. Sixth, the retrospective benchmark based on existing case materials and public datasets may limit applicability to real-world initial-visit settings.

### Future Directions and Conclusions

Future research can proceed in several directions. First, reference standards should be developed through independent annotation by larger clinical expert panels, with interrater agreement calculated to build a more reliable benchmark for rare disease specialty triage. Second, future benchmarks should evaluate patients from a given hospital or health care system using the specialty structure and triage workflow of that same institution. Third, LLM triage performance should be evaluated across multiple languages. Fourth, rare disease knowledge graphs [29], Orphanet [20], and Human Phenotype Ontology [19] can be integrated with retrieval-augmented generation [30] to improve explainability and coverage of long-tail disease-phenotype associations. Fifth, future studies should compare LLMs with established computer-assisted rare disease tools, such as Face2Gene [31] and GestaltMatcher [32], particularly in patients with facial phenotypes. Agent-based workflows integrating LLMs with visual rare disease recognition tools may further support multimodal specialty triage by combining textual clinical information with facial phenotype analysis. Sixth, human comparison studies should include larger samples and broader clinical expertise, including general practitioners, specialists, and rare disease center experts. Seventh, future studies should systematically compare paired thinking and nonthinking modes across matched model families and parameter scales, with prespecified qualitative error analysis of reasoning traces and final specialty outputs to better disentangle the effects of reasoning mode, model scale, and model-specific behavior. Finally, prospective real-world studies should assess multiturn follow-up questioning and long-term effects on triage accuracy, diagnostic delay, misdiagnosis, health care costs, and patient outcomes. Overall, LLMs show strong potential for rare disease specialty triage, and future clinical applications should position them as clinician-supervised decision-support tools with stronger evidence-tracing mechanisms to improve reliability.

## Supplementary material

10.2196/101711 Multimedia Appendix 1 This appendix includes the PubMed identifiers of publication-derived case reports, the standardized candidate specialty categories used for model outputs, exact API model identifiers and access periods, prompt templates applied across datasets, case-level clustered bootstrap confidence intervals for model accuracy, and dataset-specific performance figures showing accuracy, response time, and consistency across repeated model runs.

## Abbreviations

FGDD: Facial phenotype-Gene-Disease Dataset
LLM: large language model
