# Supplementary material for: Initial-Visit Specialty Triage in Rare Diseases Using Large Language Models: Retrospective Benchmarking Study
Source: J Med Internet Res. 2026 Jul 23;28:e101711. doi: 10.2196/101711 (PMC13394855; doi:10.2196/101711)
Supplement: Multimedia Appendix 1 [file jmir-v28-e101711-s001.docx]

# Supplementary Materials

**Supplementary Table S1.** Publication set case reports included in the study.

| **PMIDs** | | | |
| --- | --- | --- | --- |
| 32557775 | 35513831 | 37206517 | 39039767 |
| 32638640 | 35592319 | 37388486 | 39078907 |
| 32715157 | 35755060 | 37551291 | 39144887 |
| 32962641 | 35882021 | 37575808 | 39192177 |
| 33160331 | 35888564 | 37766921 | 39202561 |
| 33545995 | 35960050 | 37848221 | 39272716 |
| 33731400 | 36045763 | 37854524 | 39310618 |
| 33744411 | 36258965 | 37927735 | 39522990 |
| 33853553 | 36371175 | 37931317 | 39554391 |
| 33858421 | 36596004 | 37942396 | 39712011 |
| 33858421 | 36596004 | 38017360 | 39712766 |
| 33879466 | 36601213 | 38028072 | 39726009 |
| 34051825 | 36729003 | 38363934 | 39872528 |
| 34103306 | 36734725 | 38420100 | 39925526 |
| 34262361 | 36814168 | 38433550 | 40838094 |
| 34316810 | 36819890 | 38481933 | 40992904 |
| 34594337 | 36932345 | 38567480 | 7647418 |
| 35023661 | 37095792 | 38682093 | 8020639 |
| 35094410 | 37168520 | 38928687 |  |
| 35352601 | 37188347 | 38969060 |  |

**Supplementary Table S2.** Candidate specialty list used for rare disease specialty triage. The 30 candidate specialties based on publicly available department structures from major hospitals and academic medical centers internationally, including West China Hospital, Peking University First Hospital, Oxford University Hospitals, Karolinska University Hospital, The Johns Hopkins Hospital, Singapore General Hospital, Massachusetts General Hospital, and Mayo Clinic. We prioritized broad clinical specialties commonly present across multiple institutions and merged closely related or overlapping department names into general first-visit specialty categories. Highly regional or institution-specific centers were not included. This list was used as a unified output space for standardized model evaluation.

| **Specialty list** | | |
| --- | --- | --- |
| 1. General Internal Medicine | 11. General Surgery | 21. Hepatic Surgery |
| 2. Cardiology | 12. Orthopedic Surgery | 22. Colorectal Surgery |
| 3. Hematology | 13. Urologic Surgery | 23. Obstetrics and Gynecology |
| 4. Neurology | 14. Thoracic Surgery | 24. Pediatrics |
| 5. Endocrinology | 15. Gastrointestinal Surgery | 25. Dermatology |
| 6. Nephrology | 16. Thyroid Surgery | 26. Ophthalmology |
| 7. Gastroenterology | 17. Biliary Surgery | 27. Otolaryngology – Head and Neck Surgery |
| 8. Pulmonary and Critical Care Medicine | 18. Neurosurgery | 28. Dentistry |
| 9. Rheumatology and Immunology | 19. Vascular Surgery | 29. Infectious Diseases |
| 10. Allergy and Immunology | 20. Cardiac Surgery | 30. Breast Center |

**Supplementary Table S3**. Exact API model identifiers and API access period for the evaluated large language models.

This table lists the display names used in the manuscript, the corresponding API model identifiers used for evaluation, and the API access period during which model calls were performed.

| **LLM** | **API Model Identifier** | **API Access Date** |
| --- | --- | --- |
| Qwen3-235B-A22B-Instruct | qwen3-235b-a22b-instruct-2507 | 2026-02-01 to 2026-04-01 |
| Qwen3-235B-A22B-Thinking | qwen3-235b-a22b-thinking-2507 |  |
| Qwen3-30B-A3B-Instruct | qwen3-30b-a3b-instruct-2507 |  |
| Qwen3-30B-A3B-Thinking | qwen3-30b-a3b-thinking-2507 |  |
| LLaMA-3.1-8b | Llama-3.1-8B-Instruct |  |
| LLaMA-3.1-70b | Llama-3.1-70B-Instruct |  |
| LLaMA-3.1-405b | Llama-3.1-405B-Instruct |  |
| DeepSeek-R1 | DeepSeek-R1-0528 |  |
| DeepSeek-V3.2 | DeepSeek-V3.2 |  |
| Claude-opus-4-5 | claude-opus-4-5-20251101 |  |
| Gemini-3-pro | gemini-3-pro-preview-11-2025 |  |
| GPT-5 | gpt-5-2025-08-07 |  |
| GPT-5-mini | gpt-5-mini-2025-08-07 |  |
| GPT-5.1 | gpt-5.1-2025-11-13 |  |

**Supplementary Table S4**. Prompt templates used across datasets

| Dataset | Prompt |
| --- | --- |
| RareBench HMS, RareBench LIRICAL, RareBench MME, FGDD | Based on the patient’s phenotype information, which specialist department is most appropriate?  "Patient phenotype information": {phenotype information}  Department list:  {department list} |
| Publication set | Based on the patient’s following information, which specialist department is most appropriate?  "Patient Demographics": {patient_demographics},  "Presenting Complaint and Current Clinical Manifestations": {clinical_manifestations},  "Medical History": {medical_history},  "Family Medical History": {family_history}  Department list:  {department list} |

**Supplementary Table S5.** Case-level clustered bootstrap 95% confidence intervals for model accuracy. Accuracy SD was calculated across five independent runs. Bootstrap 95% confidence intervals were calculated using a case-level clustered bootstrap, in which cases were resampled with replacement and the five repeated runs for each sampled case were kept together.

| **LLM** | **Accuracy, mean ± SD** | **Bootstrap 95% CI** |
| --- | --- | --- |
| Qwen3-235B-A22B-Instruct | 0.6960 ± 0.0100 | [0.6617, 0.7294] |
| Qwen3-235B-A22B-Thinking | 0.6671 ± 0.0064 | [0.6353, 0.6992] |
| Qwen3-30B-A3B-Instruct | 0.6932 ± 0.0083 | [0.6595, 0.7266] |
| Qwen3-30B-A3B-Thinking | 0.4378 ± 0.0116 | [0.4105, 0.4652] |
| LLaMA-3.1-8B | 0.6238 ± 0.0149 | [0.5901, 0.6569] |
| LLaMA-3.1-70B | 0.6359 ± 0.0083 | [0.6013, 0.6703] |
| LLaMA-3.1-405B | 0.6706 ± 0.0118 | [0.6366, 0.7033] |
| DeepSeek-R1 | 0.6429 ± 0.0053 | [0.6079, 0.6766] |
| DeepSeek-V3.2 | 0.6614 ± 0.0083 | [0.6280, 0.6941] |
| Claude-opus-4-5 | 0.7141 ± 0.0013 | [0.6795, 0.7485] |
| Gemini-3-pro | 0.7049 ± 0.0092 | [0.6703, 0.7390] |
| GPT-5 | 0.6839 ± 0.0069 | [0.6486, 0.7186] |
| GPT-5-mini | 0.6890 ± 0.0081 | [0.6547, 0.7231] |
| GPT-5.1 | 0.6948 ± 0.0011 | [0.6595, 0.7281] |


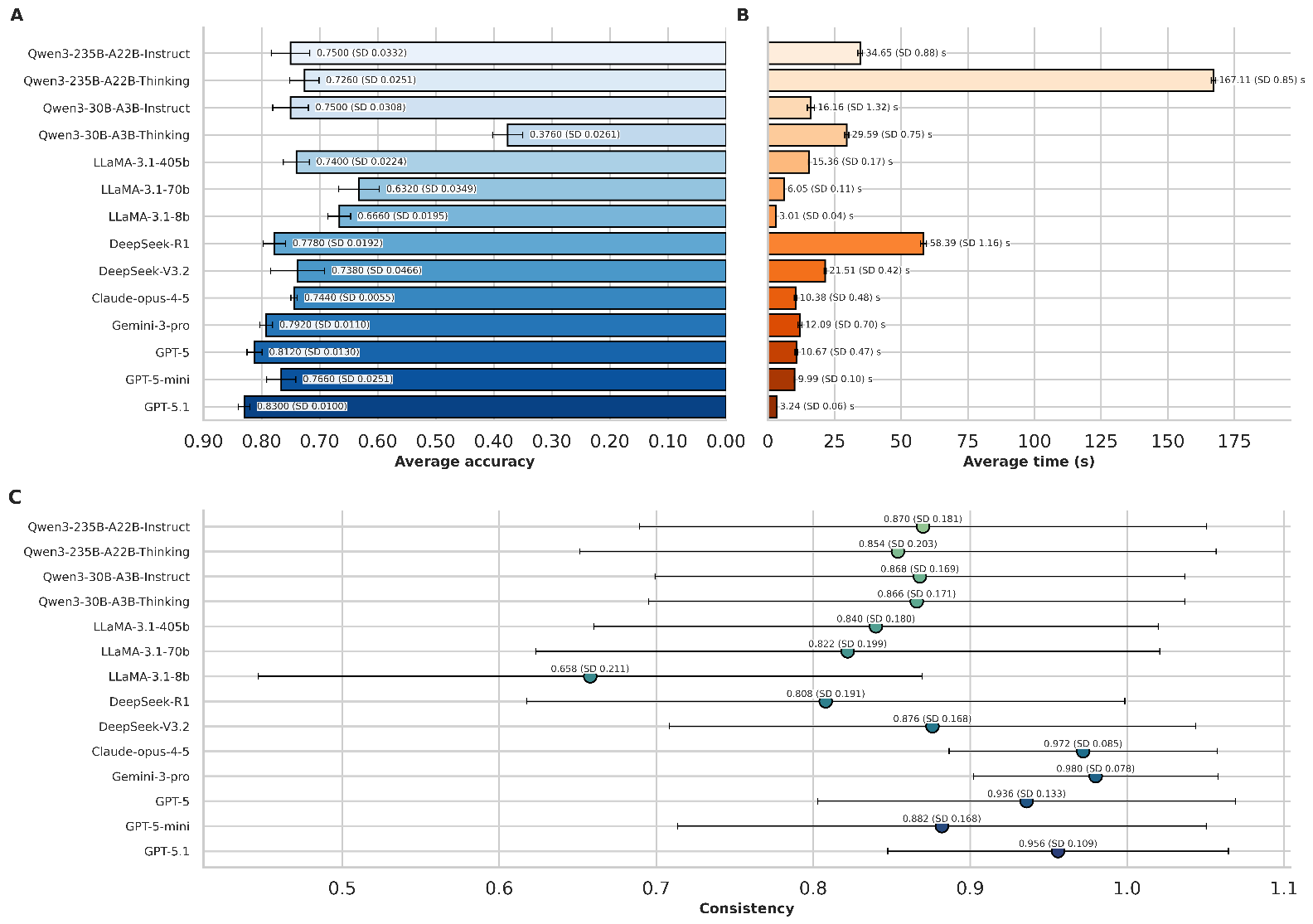


**Supplementary Figure S1.** Overall performance of LLMs for rare disease specialty triage in FGDD. (a) Average accuracy across five independent runs. (b) Average response time per case. (c) Average consistency across five repeated runs.


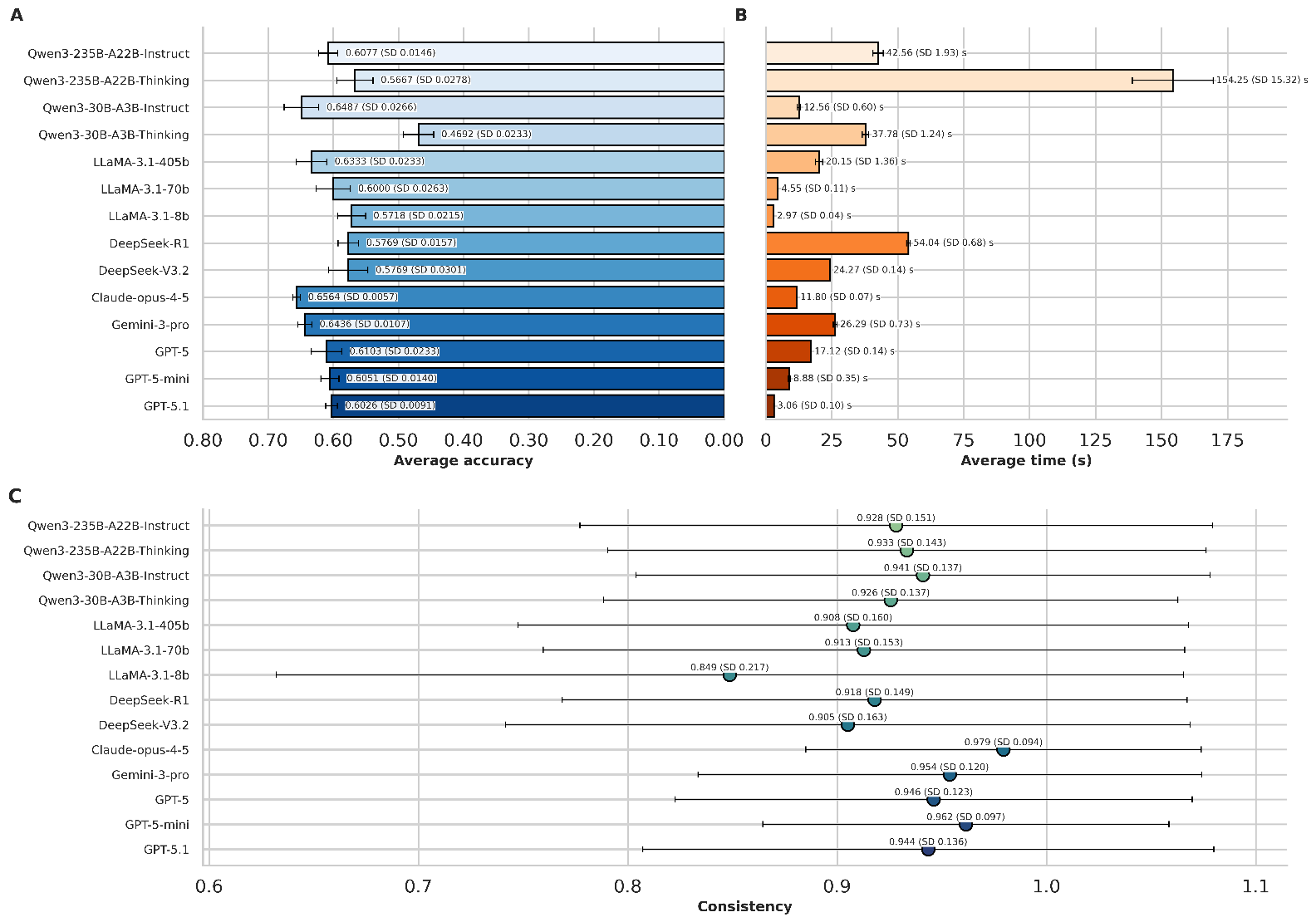


**Supplementary Figure S2.** Overall performance of LLMs for rare disease specialty triage in Publication set. (a) Average accuracy across five independent runs. (b) Average response time per case. (c) Average consistency across five repeated runs.


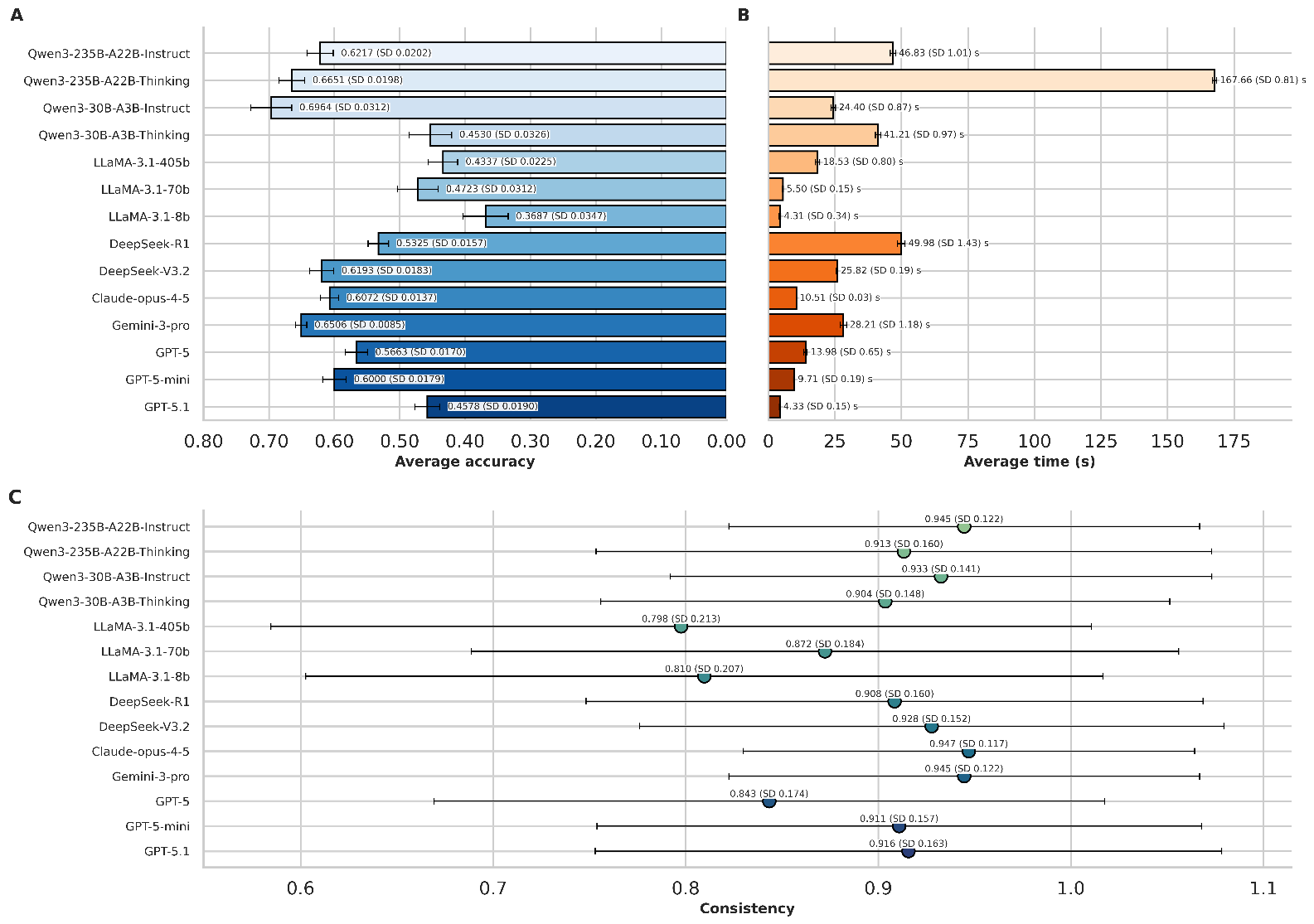


**Supplementary Figure S3.** Overall performance of LLMs for rare disease specialty triage in Rarebench_HMS. (a) Average accuracy across five independent runs. (b) Average response time per case. (c) Average consistency across five repeated runs.


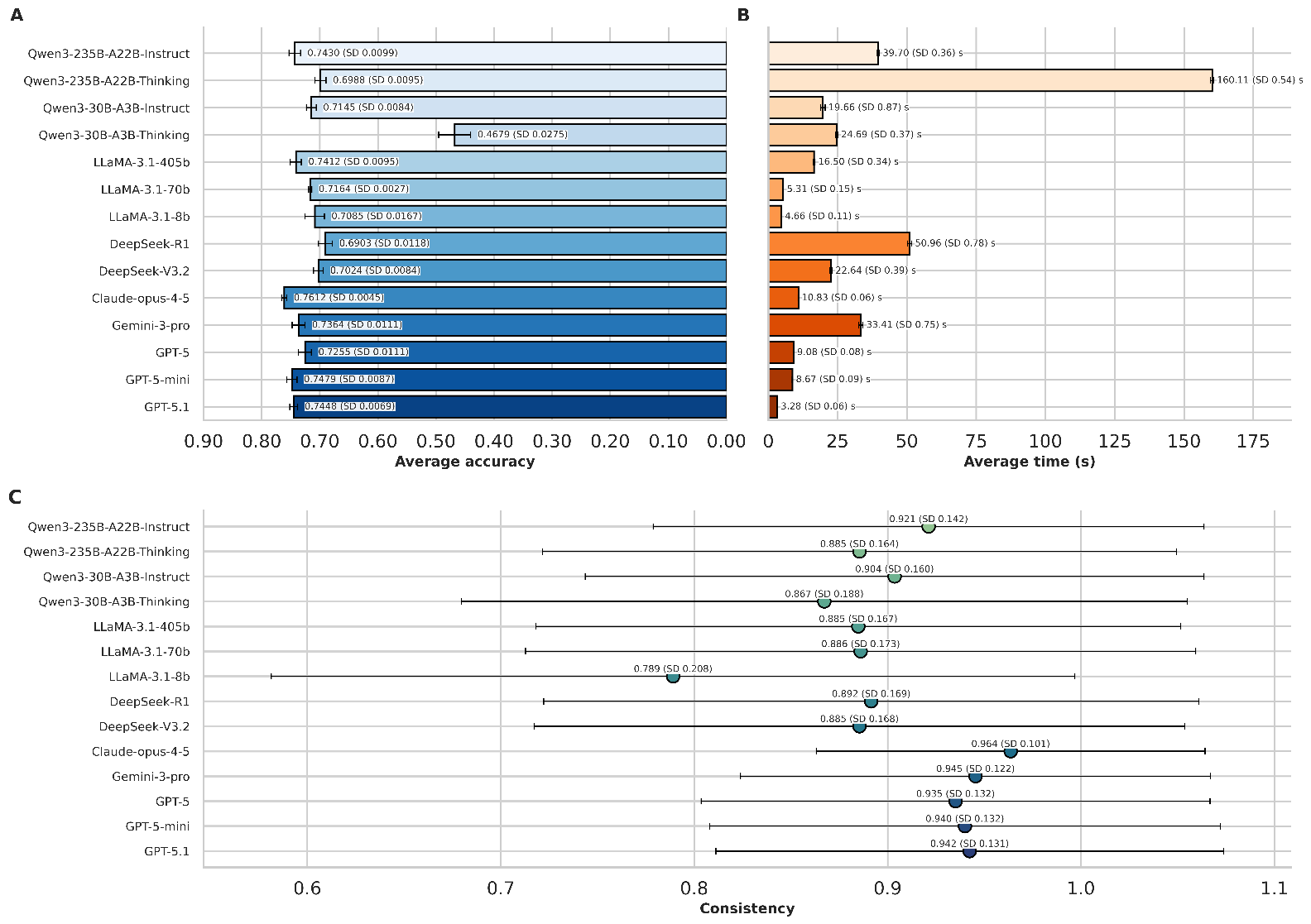


**Supplementary Figure S4.** Overall performance of LLMs for rare disease specialty triage in Rarebench_LIRICAL. (a) Average accuracy across five independent runs. (b) Average response time per case. (c) Average consistency across five repeated runs.


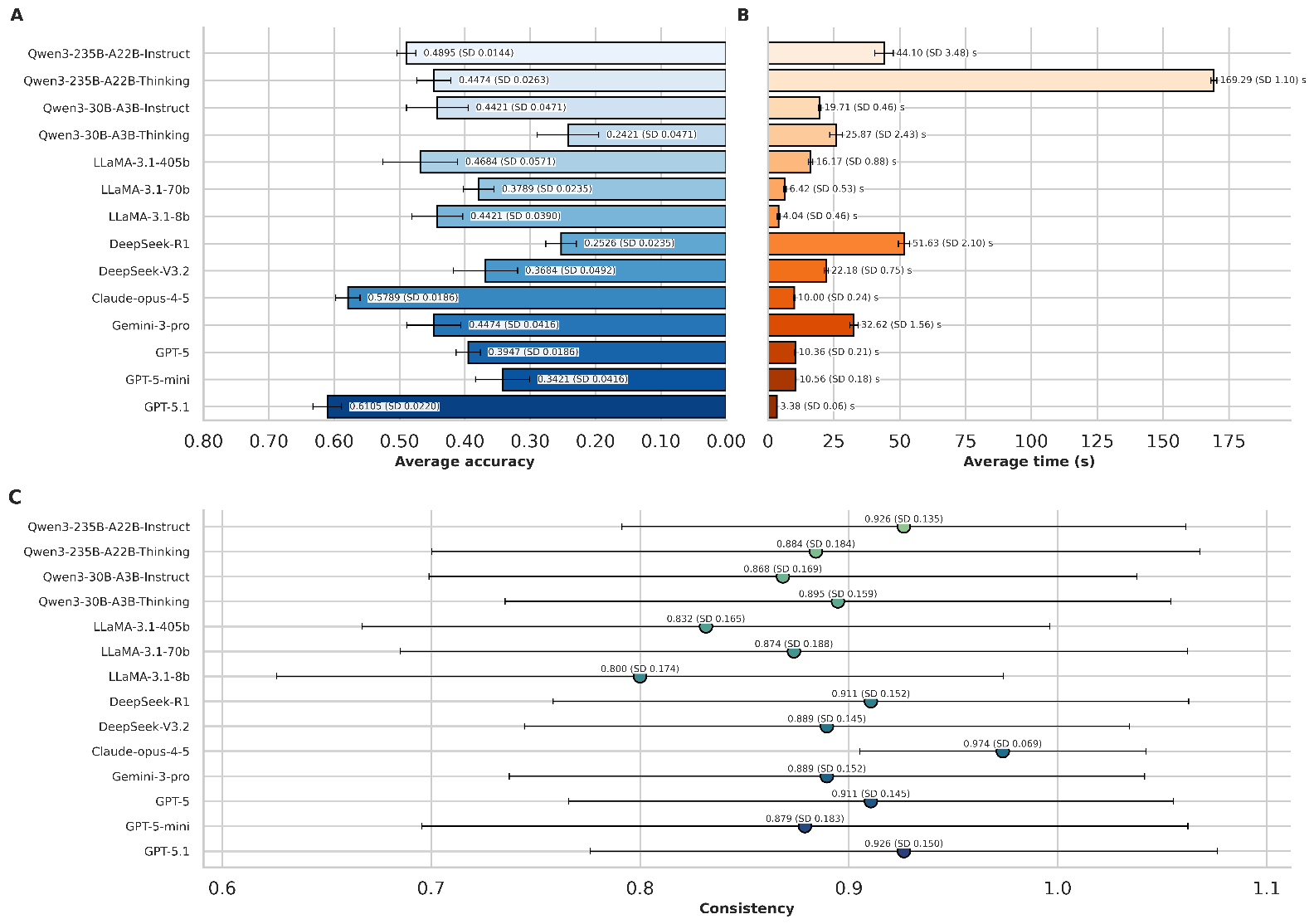


**Supplementary Figure S5.** Overall performance of LLMs for rare disease specialty triage in Rarebench_MME. (a) Average accuracy across five independent runs. (b) Average response time per case. (c) Average consistency across five repeated runs.
